# Distinguishing the Leading Agents in Classification Problems Using the Entropy-Based Metric

**DOI:** 10.3390/e26040318

**Published:** 2024-04-05

**Authors:** Evgeny Kagan, Irad Ben-Gal

**Affiliations:** 1 Department of Industrial Engineering, Ariel University, Ariel 4076414, Israel; 2 Department of Industrial Engineering, Tel Aviv University, Tel Aviv 6997801, Israel; bengal@tau.ac.il

**Keywords:** leading agents, classification, entropy, Rokhlin metric

## Abstract

The paper addresses the problem of distinguishing the leading agents in the group. The problem is considered in the framework of classification problems, where the agents in the group select the items with respect to certain properties. The suggested method of distinguishing the leading agents utilizes the connectivity between the agents and the Rokhlin distance between the subgroups of the agents. The method is illustrated by numerical examples. The method can be useful in considering the division of labor in swarm dynamics and in the analysis of the data fusion in the tasks based on the wisdom of the crowd techniques.

## 1. Introduction

The behavior of the group of autonomous agents includes a variety of physical and cognitive actions, like collective motion and cooperative decision-making. Each of these actions depends on the individual abilities of the agents, on the division of the agents among the teams, and on the division of labor between the agents and the teams.

To simplify control of the teams’ and the group’s activities, some of the agents are often determined as leaders, whose task is to influence the other agents and promote them to fulfill the mission. Such procedures are widely known as elections in distributed systems, which are considered both in social and political processes and in different contexts of computer science and robots’ control [1,2,3].

Given a group of communicating agents, elections are conducted as follows: The agents consider the candidate agents, and after deliberations, they identify and label a certain agent as the leader. Then, the elected leader influences the other agents and coordinates their activities [1]. Certainly, each team in the group can elect its leader, and then these leaders elect the leader of the group, which results in hierarchical control in the system.

The process that is close to the election of the leader is the selection of the leader [4,5,6,7]. In this process, the leader is selected according to the known characteristics of the agents and their correspondence with the characteristics required for fulfilling the mission of the group. Usually, after the section of the leader, the other agents are considered followers. Note that, in contrast to the election of the leader, the leader selection is not necessarily conducted by the agents but can be processed by the central coordinator or controller of the group.

Now, assume that the group leader or the team leader already exists. Then rises an inverse problem—to distinguish the leaders in the distributed system. Namely, given a group of communicating agents, it is required to identify the leaders, which are the agents who mostly influence the other agents in the group.

In the paper, the problem of distinguishing the leading agents is considered in the context of the classification problem [8,9]. In such a classification, it is assumed that the agents have different levels of expertise, and the cooperative classification is obtained by a certain version of plurality voting.

The most popular method of avoiding the influence of non-competent agents uses the weighted opinions of the agents. This method was implemented in the well-known Dawid–Skene algorithm [10,11], which iterates the expectation of the correct choice with respect to the agents’ expertise and maximizes the likelihood of the agents’ expertise with respect to the expected correct choice.

Another approach to selecting the competent agents was implemented in the algorithms [12,13], which are based on the similarities of the agents’ classifications, and in the algorithm [14], where the competent agents are selected using the expectation bias [15].

After selecting the subgroup of competent agents, the resulting classification is obtained using the opinions of these agents and ignoring the opinions of non-competent agents.

However, studies in social psychology [16], which can be traced back to the well-known experiments by Asch [17,18], demonstrate that the opinion of the group member is highly influenced by the opinions of the other members of the group. Consequently, competent agents are not necessarily the most influential or leading agents. Together with that, it is reasonable to assume that the leader must be competent in certain fields and be elected on the basis of this competence (see the 11th rule by Peterson [19]).

In the paper, we suggest a method of distinguishing the leaders in the group. The method considers the connectivity between the agents and creates the subgroups by maximizing the distances between the partitions formed by these subgroups.

Note that the elections in the group are internal processes in which the leaders are identified following certain criteria known to the agents, while distinguishing the leaders is an external operation in which the agents are characterized by their relations with the other agents. Thus, the distinguished leaders can differ from the elected leaders: elections specify who will govern, and distinguishing specifies who must govern.

The rest of the paper is organized as follows: Section 2 includes a formulation of the problem, and Section 3 considers an example that clarifies the problems of classification, distinguishing the competent agents, and distinguishing the leading agents. Section 4 presents a suggested solution based on the connectivity between the agents and the Rokhlin distance between the agents’ subgroups. Section 5 presents the methods of distinguishing the experts, and Section 6 considers two examples that illustrate the relationship between the group of experts and the group of leaders. Section 7 concludes the discourse.

## 2. Problem Formulation

The main problem considered in the paper is the problem of distinguishing the leaders in the group of agents. As indicated above, this problem differs from the problem of electing the leader and requires consideration of communication between the agents.

We consider the problem in the context of the classification of given items by a group of agents, where some of the agents are experts in the field of classification and others are deletants.

Consideration of these two problems gives rise to the third problem, which is the problem of relations between the group of leaders and the group of experts.

### 2.1. Distinguishing the Leaders

Let $A=\left\{a_{1},a_{2},\ldots ,a_{l}\right\}$ be a group of communicating agents conducting a certain common mission. Communication between the agents is defined by the directed graph $G=\left(V,E\right)$ in which the vertices v∈V are associated with the agents and the edges e∈E are defined by the adjacency matrix $R={\left(r_{ij}\right)}_{l\times l}$ such that $r_{ij}=1$ if agent $a_{i}$ communicates with agent $a_{j}$ and $r_{ij}=0$ otherwise.

The problem of distinguishing the leaders in group A is formulated as follows: Given the adjacency matrix R, it is required to recognize a subgroup $A^{*}\subset A$ of the agents such that the agents $a^{*}\in A^{*}$ have maximal influence in the group A.

Consideration of the agent’s influence in the group is based on the following intuition inspired by the flows on graphs (see, e.g., [20]). Assume that each vertex v∈V in the graph G is a source of unique colored flow and that the color of the vertex is a mix of its own color and the colors of the incoming flows. Then, the influence of the agent is associated with the impact of its vertex on the colors of the other vertices in the graph G.

The vertices $v^{*}\in V^{*}\subset V$ associated with the leading agents $a^{*}\in A^{*}$ are called the leading vertices. The leading agent such that its vertex in the graph G has no predecessors is called a dictator, and the leading agent whose vertex is a separating vertex (see, e.g., [21]) is called a monarch.

### 2.2. Distinguishing the Experts

Let $X=\left\{x_{1},\ldots ,x_{n}\right\}$ be a set of n items, which represent certain objects, concepts, or symbols. Classification problem requires to label the items $x_{i}\in X$, i=1,2,…,n, by m labels, 1<m<n, such that each item is labeled by a single label.

Formally, the problem is to distribute the items $x_{i}\in X$, i=1,2,…,n, over m sets $C_{1},C_{2},\ldots ,C_{m}$, 1<m<n, called classes, such that each item $x_{i}$ is included only in one class $C_{j}$ and that there is no item which is not included in some classes. Then, the resulting classification is the partition $\gamma =\left\{C_{1},C_{2},\ldots ,C_{m}\right\}$ of the set X, where $C_{j}\subset X$, j=1,…,m, $C_{j^{\prime }}\cap C_{j^{\prime \prime }}=\emptyset$ for $j^{\prime }\neq j^{\prime \prime }$, and $\overset{m}{\underset{j=1}{⋃}}C_{j}=X$.

If classification is conducted by a single agent, then the resulting classification γ depends on the competence of this agent. The quality of classification is defined by the difference between the classification γ and the correct classification $\overset{ˇ}{\gamma }$. Certainly, the correct classification $\overset{ˇ}{\gamma }$ is not available to the agent and is used for testing the classification methods.

Now assume that the classification is conducted by the indicated above group $A=\left\{a_{1},a_{2},\ldots ,a_{l}\right\}$ of agents where each agent $a_{k}\in A$, k=1,2,…,l, provides classification represented by the partition $\gamma_{k}=\left\{C_{k,1},C_{k,2},\ldots ,C_{k,m}\right\}$. By the general assumption of “the wisdom of the crowd” techniques [8,9], some combination of the agents’ classification will provide classification γ, which is as close as possible to the correct classification $\overset{ˇ}{\gamma }$.

Then, the problem is to aggregate the agents’ partitions $\gamma_{k}$ into a single partition γ such that it, as best as possible, represents the correct partition $\overset{ˇ}{\gamma }$.

The simplest method of creating the partition γ is plurality voting. By this method, the item x is included in the class C, which was chosen by most agents a∈A (the ties are broken randomly). Despite its popularity, this method strongly depends on the competence of the agents, such that non-competent agents can influence the resulting classification.

To avoid such influence, in more sophisticated methods [10,11,12,13,14], the problem is divided into two stages. First, using the agents’ classifications $\gamma_{k}$, k=1,2,…,l, the agents competent in certain classes are distinguished, and second, the resulting classification γ is obtained by aggregation of the classes provided by the competent agents.

### 2.3. Relationship between the Leaders and the Experts

Finally, assume that the group $A=\left\{a_{1},a_{2},\ldots ,a_{l}\right\}$ of l communicating agents classifies the set $=\left\{x_{1},\ldots ,x_{n}\right\}$ of n items to m classes $C_{1},C_{2},\ldots ,C_{m}$, 1<m<n.

In addition, assume that the group A of the agents includes a non-empty subgroup $A^{*}\subset A$ of leaders and non-empty subgroup $A^{\prime }\subset A$ of experts.

Then, the problem is to check whether there exists a relationship between the set of leaders $A^{*}$ and the set $A^{\prime }$ of experts, and if it exists, what this relationship is.

We assume that in real-world situations, such a relationship is possible, and the leaders are assumed to be experts in at least one class. The problem is to confirm or withdraw this hypothesis.

## 3. Illustrative Example

**Figure 1:**
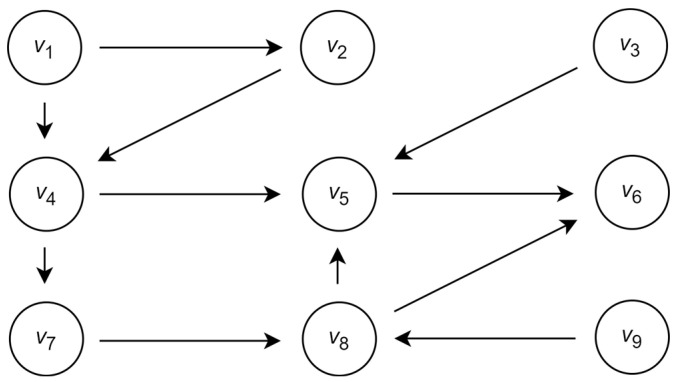
Example of the graph defining communication between the agents.

Assume that the group A includes l=9 communicating agents and that communication between the agents is defined by the directed graph $G=\left(V,E\right)$, where the vertices v∈V are associated with the agents and the edges e∈E specify the communication between the corresponding agents. The graph G is shown in Figure 1.

The sets of input and output vertices in this graph are presented in Table 1.

As indicated above, in consideration of the agents’ impacts, each vertex of the graph G is considered a source of the unique colored flow. The color absorbed by the vertex is a mix of its own color and the colors of the incoming flows. The impact of the agent is considered to be the impact of its vertex on the color of the other vertices. Then, intuitively, the vertex with a maximal number of predecessors and successors is associated with the agents with maximal influence.

Following the cardinality of the sets of input and output vertices, it can be supposed that the set of vertices associated with the candidates to the leading agents is $V^{c}=\left\{v_{1},v_{4},v_{5},v_{8}\right\}$ such that $a_{1}$ is a dictator and $a_{4}$ is a monarch. In addition, because of the maximal number of predecessors, intuitively, vertex $v_{6}$ can also be considered a candidate for the leading vertex.

Assume that the agents a from the group A distribute n=12 items x from the set X over m=4 classes C. The results of the classification are shown in Table 2.

In the table, the first agent $a_{1}$ included the item $x_{1}$ into the class $C_{2}$, the item $x_{2}$ into the class $C_{1}$, the item $x_{3}$ into the class $C_{3}$ and so on, such that the partition $\gamma_{1}$ created by the first agent is

$$\gamma_{1}=\left\{\left\{x_{2},x_{6},x_{8}\right\},\left\{x_{1}\right\},\left\{x_{3},x_{9},x_{10},x_{12}\right\},\left\{x_{4},x_{5},x_{7},x_{11}\right\}\right\}.$$

The second agent $a_{2}$ included the item $x_{1}$ into the class $C_{2}$, the item $x_{2}$ into the class $C_{3}$, the item $x_{3}$ into the class $C_{2}$ and so on, such that the second agent is

$$\gamma_{2}=\left\{\emptyset ,\left\{x_{1},x_{3},x_{10}\right\},\left\{x_{2},x_{6},x_{7},x_{8},x_{9},x_{11},x_{12}\right\},\left\{x_{4},x_{5}\right\}\right\},$$

and so on. Correct classification is represented by the following partition:

$$\overset{ˇ}{\gamma }=\left\{\left\{x_{2},x_{6},x_{8}\right\},\left\{x_{1}\right\},\left\{x_{3},x_{9},x_{10},x_{12}\right\},\left\{x_{4},x_{5},x_{7},x_{11}\right\}\right\},$$

and the partition obtained by the plurality voting is

$$\gamma_{Pl}=\left\{\left\{x_{2},x_{3},x_{6},x_{10}\right\},\left\{x_{1},x_{8}\right\},\left\{x_{11},x_{12}\right\},\left\{x_{4},x_{7},x_{9}\right\}\right\},$$

It is seen that the classifications $\gamma_{k}$ provided by the agents $a_{k}$, k=1,2,…,l, are rather far from the correct classification $\overset{ˇ}{\gamma }$ as well as the aggregated classification $\gamma_{Pl}$ obtained by plurality voting.

However, some agents created certain classes that are equivalent to the classes in the correct classification, namely,

- agent $a_{1}$—class $C_{1}=\left\{x_{2},x_{6},x_{8}\right\}$,
- agent $a_{2}$—class $C_{2}=\left\{x_{1},x_{3},x_{10}\right\}$,
- agent $a_{3}$—classes $C_{1}=\left\{x_{2},x_{6},x_{8}\right\}$ and $C_{2}=\left\{x_{1},x_{3},x_{10}\right\}$,
- agent $a_{4}$—class $C_{3}=\left\{x_{4},x_{5},x_{11}\right\}$,
- agent $a_{5}$—class $C_{4}=\left\{x_{7},x_{9},x_{12}\right\}$,
- agent $a_{6}$—classes $C_{3}=\left\{x_{4},x_{5},x_{12}\right\}$ and $C_{4}=\left\{x_{7},x_{9},x_{12}\right\}$.

The other agents $a_{7}$, $a_{8}$ and $a_{9}$ provided completely erroneous classifications, where despite the correct classification of some items, all obtained classes differ from the classes appearing in the correct classification. Then, aggregating the appropriate classes from the classifications created by the agents $a_{1},\ldots ,a_{6}$ and avoiding classifications created by the agents $a_{7},\ldots ,a_{9}$, provides correct classification $\gamma =\overset{ˇ}{\gamma }$.

In the considered case of random classifications and relations between the agents, a possible set of leaders is $A^{*}=\left\{a_{1},a_{4},a_{5},a_{8}\right\}$ and possible set of experts is $A^{\prime }=\left\{a_{1},a_{2},a_{3},a_{4},a_{5},a_{6}\right\}$, which means that there is no clear relationship between these sets. However, since in real-world situations, the leader must be competent in at least one field of knowledge, the absence of such a relationship is not obvious, and its consideration is reasonable.

## 4. Distinguishing the Group of Leaders Using the Entropy-Based Metric

Let $A=\left\{a_{1},a_{2},\ldots ,a_{l}\right\}$ be a group of agents and $G=\left(V,E\right)$ be the directed graph representing communication between the agents such that the vertices v∈V are associated with the agents and the edges $e=\left(v_{i},v_{j}\right)\in E$ define communication between the agents $a_{i},a_{j}\in A$, i,j=1,2,…,l.

Denote by $\overset{⃐}{u}\left(v,\xi \right)\in V$ a predecessor of the vertex v∈V such that the shortest path from $\overset{⃐}{u}\left(v,\xi \right)$ to v in the graph G is of the length ξ, and by $\overset{⃑}{u}\left(v,\xi \right)\in V$ a successor of the vertex v∈V such that the shortest path from v to $\overset{⃑}{u}\left(v,\xi \right)$ in the graph G is of the length ξ. In particular, $\overset{⃐}{u}\left(v,1\right)=\overset{⃐}{u}\left(v\right)$ is a direct predecessor of v and $\overset{⃑}{u}\left(v,1\right)=\overset{⃑}{u}\left(v\right)$ is a direct successor of v. For completeness, we also say that $\overset{⃐}{u}\left(v,0\right)=\overset{⃑}{u}\left(v,0\right)=v$.

It is clear that each v∈V and all its predecessors are the predecessors of each successor of v, and each v∈V and all its successors are the successors of each predecessor of v.

For a vertex v∈V, denote by $\overset{⃐}{U}\left(v,\xi \right)$, the set of its predecessors $\overset{⃐}{u}\left(v,\xi \right)$ and by $\overset{⃑}{U}\left(v,\xi \right)$ the set of all its successors $\overset{⃑}{u}\left(v,\xi \right)$. The set of direct predecessors is denoted by $\overset{⃐}{U}\left(v,1\right)=\overset{⃐}{U}\left(v\right)$ and the set of direct successors is denoted by $\overset{⃑}{U}\left(v,1\right)=\overset{⃑}{U}\left(v\right)$.

Given a graph G, let $\overset{⃐}{U}\left(v\right)$ be the set of direct predecessors of the vertex v, $\overset{⃐}{U}\left(\overset{⃐}{u}\left(v\right)\right)$ be the sets of direct predecessors of the vertices $\overset{⃐}{u}\left(v\right)\in \overset{⃐}{U}\left(v\right)$, $\overset{⃐}{U}\left(\overset{⃐}{u}\left(\overset{⃐}{u}\left(v\right)\right)\right)$ be the sets of direct predecessors of the vertices $\overset{⃐}{u}\left(\overset{⃐}{u}\left(v\right)\right)\in \overset{⃐}{U}\left(\overset{⃐}{u}\left(v\right)\right)$ and so on, up to, but not including, the set that already appears among the sets of direct predecessors obtained at the previous steps.

Similarly, let $\overset{⃑}{U}\left(v\right)$ be the set of direct successors of the vertex v, $\overset{⃑}{U}\left(\overset{⃑}{u}\left(v\right)\right)$ be the sets of direct successors of the vertices $\overset{⃑}{u}\left(v\right)\in \overset{⃑}{U}\left(v\right)$, $\overset{⃑}{U}\left(\overset{⃑}{u}\left(\overset{⃑}{u}\left(v\right)\right)\right)$ be the sets of direct successors of the vertices $\overset{⃑}{u}\left(\overset{⃑}{u}\left(v\right)\right)\in \overset{⃑}{U}\left(\overset{⃑}{u}\left(v\right)\right)$ and so on, up to, but not including, the set that already appears among the sets of direct successors obtained at the previous steps.

Finally, for the vertex v, let us form the predecessors’ tree $\overset{⃐}{T}\left(v\right)$ and the successors’ tree $\overset{⃑}{T}\left(v\right)$. In the tree $\overset{⃐}{T}\left(v\right)$, the root is associated with the set $\overset{⃐}{U}\left(v\right)$ and the leaves at their levels are associated with the sets $\overset{⃐}{U}\left(\overset{⃐}{u}\left(v\right)\right)$, $\overset{⃐}{U}\left(\overset{⃐}{u}\left(\overset{⃐}{u}\left(v\right)\right)\right)$ and so on, respectively. Similarly, in the $\overset{⃑}{T}\left(v\right)$, the root is associated with the set $\overset{⃑}{U}\left(v\right)$ and the leaves at their levels are associated with the sets $\overset{⃑}{U}\left(\overset{⃑}{u}\left(v\right)\right)$, $\overset{⃑}{U}\left(\overset{⃑}{u}\left(\overset{⃑}{u}\left(v\right)\right)\right)$ and so on.

**Figure 2:**
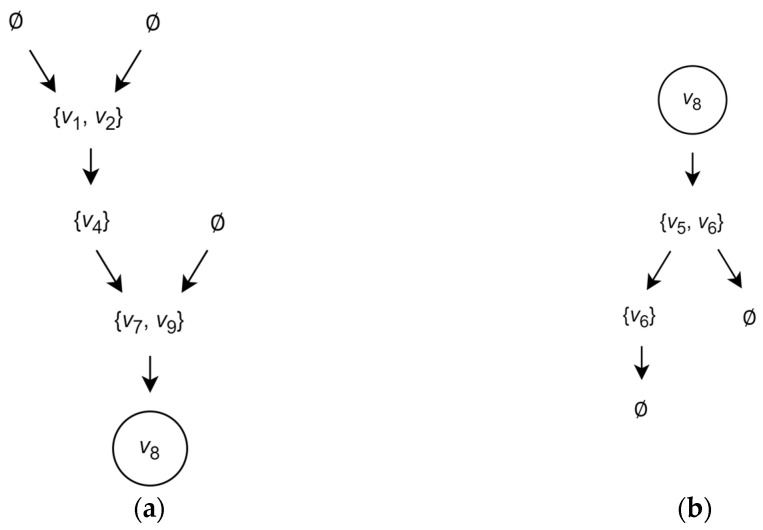
Example of the predecessors’ and successors’ trees: (**a**) the predecessors’ tree $\overset{⃐}{T}\left(v_{8}\right)$ and (**b**) the successors’ tree $\overset{⃑}{T}\left(v_{8}\right)$ of the vertex $v_{8}$ in the graph shown in Figure 1.

For illustration, the predecessors’ tree $\overset{⃐}{T}\left(v_{8}\right)$ and the successors’ tree $\overset{⃑}{T}\left(v_{8}\right)$ of the vertex $v_{8}$ in the graph G are shown in Figure 2.

The sets associated with the leaves of the trees $\overset{⃐}{T}\left(v\right)$ and $\overset{⃑}{T}\left(v\right)$ form, respectively, the predecessor cover $\overset{⃐}{\tau }\left(v\right)$ and the successor cover $\overset{⃑}{\tau }\left(v\right)$ of certain subsets of the set V of vertices.

For example, the predecessor and successor covers of the vertex $v_{8}$ are $\overset{⃐}{\tau }\left(v_{8}\right)=\left\{\left\{v_{1},v_{2}\right\},\left\{v_{4}\right\},\left\{v_{7},v_{9}\right\}\right\}$ and $\overset{⃑}{\tau }\left(v_{8}\right)=\left\{\left\{v_{5},v_{6}\right\},\left\{v_{6}\right\}\right\}$.

The predecessor cover $\overset{⃐}{\tau }\left(V^{\prime }\right)$ of the subset $V^{\prime }\subset V$ of vertices is a set

$$\overset{⃐}{\tau }\left(V^{\prime }\right)={⋃}_{v\in V^{\prime }}\overset{⃐}{\tau }\left(v\right)$$

of the predecessor covers $\overset{⃐}{\tau }\left(v\right)$ of the vertices $v\in V^{\prime }$, and the successor cover $\overset{⃑}{\tau }\left(V^{\prime \prime }\right)$ of the subset $V^{\prime \prime }\subset V$ of vertices is a set

$$\overset{⃑}{\tau }\left(V^{\prime \prime }\right)={⋃}_{v\in V^{\prime \prime }}\overset{⃑}{\tau }\left(v\right)$$

of the successor covers $\overset{⃐}{\tau }\left(v\right)$ of the vertices $v\in V^{\prime \prime }$.

For example, for the indicated above set $V^{c}=\left\{v_{1},v_{4},v_{5},v_{8}\right\}$ of vertices, the predecessor and successor covers are $\overset{⃐}{\tau }\left(V^{c}\right)=\left\{\left\{v_{1},v_{2}\right\},\left\{v_{3},v_{4},v_{8}\right\},\left\{v_{4}\right\},\left\{v_{7},v_{9}\right\}\right\}$ and $\overset{⃑}{\tau }\left(V^{c}\right)=\left\{\left\{v_{2},v_{4}\right\},\left\{v_{4}\right\},\left\{v_{5},v_{6}\right\},\left\{v_{5},v_{7}\right\},\left\{v_{8}\right\}\right\}$.

Then, we say that the subset $V^{*}\subset V$ is a set of leading vertices if the distance $d\left(\overset{⃐}{\tau }\left(V^{*}\right),\overset{⃑}{\tau }\left(V^{*}\right)\right)$ between its predecessor cover $\overset{⃐}{\tau }\left(V^{*}\right)$ and successor cover $\overset{⃑}{\tau }\left(V^{*}\right)$ is maximal over all possible subsets of the set V.

The agents $a^{*}$ associated with the leading vertices $v^{*}\in V^{*}$ are called the leaders and the group $A^{*}\subset A$ of leading agents is called the leading group.

Distance $d\left(\overset{⃐}{\tau }\left(V^{\prime }\right),\overset{⃑}{\tau }\left(V^{\prime \prime }\right)\right)$ between the covers $\overset{⃐}{\tau }\left(V^{\prime }\right)$ and $\overset{⃑}{\tau }\left(V^{\prime \prime }\right)$ can be calculated using different methods. Here, we suggest the distance measure, which is based on the Rokhlin metric [22]. Since in the considered task, the main stress is on the communication between the agents and on the classification of the data items, the use of such an entropy-based metric is reasonable. Together with that, since the suggested method deals with formal sets of vertices in the graph, the other measures, e.g., the Ornstein distance [23,24], can be applied. For a comparison between the Rokhlin distance and the Ornstein distance, see [25]. Note that both Rokhlin and Ornstein metrics require the defined probability measure on the sets; if such a probability does not exist, then the normalized Hamming distance [12] can be used.

The Rokhlin metric is defined as follows. Let $\left(\Omega ,Q,p\right)$ be a probability space with a probability measure p on Ω, and let $\alpha =\left\{Q|Q\in Q\right\}$, $Q_{i}\cap Q_{j}=\emptyset$, i≠j, ${⋃}_{Q\in \alpha }Q=\Omega$, be a partition of Ω. The entropy of the partition α is the value

$$H\left(\alpha \right)=-\sum_{Q\in \alpha }p\left(Q\right)\log p\left(Q\right),$$

where log is base 2, and it is assumed that $p\left(\emptyset \right)\log p\left(\emptyset \right)=0\log0=0$. In addition, let $\beta =\left\{R|R\in Q\right\}$, $R_{i}\cap R_{j}=\emptyset$, i≠j, ${⋃}_{R\in \beta }R=\Omega$, be another partition of Ω. Then the conditional entropy of partition α given partition β is the value

$$H\left(\alpha |\beta \right)=-\sum_{R\in \beta }\sum_{Q\in \alpha }p\left(Q,R\right)\log p\left(Q|R\right),$$

where $p\left(Q,R\right)=p\left(Q\cap R\right)$ and $p\left(Q|R\right)=\frac{p\left(Q\cap R\right)}{p\left(R\right)}$.

The Rokhlin metric [22], which defines the distance between partitions α and β is a sum

$$d_{R}\left(\alpha ,\beta \right)=H\left(\alpha |\beta \right)+H\left(\beta |\alpha \right),$$

For basic properties of this metric and its role in dynamical systems theory, see [26,27]; for additional properties and comparison with the Ornstein metric, see [25].

To apply this metric for measuring the distance between the covers $\overset{⃐}{\tau }\left(V^{\prime }\right)$ and $\overset{⃑}{\tau }\left(V^{\prime \prime }\right)$, note again that each of these sets does not necessarily cover the set of vertices V, but the subsets ${⋃}_{Q\in \overset{⃐}{\tau }\left(V^{\prime }\right)}Q\subset V$ and ${⋃}_{R\in \overset{⃐}{\tau }\left(V^{\prime \prime }\right)}R\subset V$ of this set. Then, let us add to each of these sets the set which completes it to the cover of V.

Namely, the set $\overset{⃐}{\tau }\left(V^{\prime }\right)$ is completed with the set $Q^{\prime }=V\backslash {⋃}_{Q\in \overset{⃐}{\tau }\left(V^{\prime }\right)}Q$ and the set $\overset{⃑}{\tau }\left(V^{\prime \prime }\right)$ is completed with the set $R^{\prime }=V\backslash {⋃}_{R\in \overset{⃑}{\tau }\left(V^{\prime \prime }\right)}R$. As a result, the sets

$${\overset{⃐}{\tau }}^{\prime }\left(V^{\prime }\right)=\overset{⃐}{\tau }\left(V^{\prime }\right)\cup \left\{Q^{\prime }\right\}\;\mathrm{and}\;{\overset{⃑}{\tau }}^{\prime }\left(V^{\prime \prime }\right)=\overset{⃑}{\tau }\left(V^{\prime \prime }\right)\cup \left\{R^{\prime }\right\}$$

cover the set V of vertices.

Then, it is required to define the probability measure $p:V\to \left[0,1\right]$ on the set of vertices. Since there is no additional information about the agents, we assume that

$$p\left(v\right)=\frac{1}{\#V}$$

for each vertex v∈V, and

$$p\left(Q\right)=\sum_{v\in Q}p\left(v\right)=\frac{\#Q}{\#V}$$

for each subset Q⊂V of vertices.

For the conditional entropy, we have

$$\begin{array}{ll} H\left({\overset{⃐}{\tau }}^{\prime }\left(V^{\prime }\right)|{\overset{⃑}{\tau }}^{\prime }\left(V^{\prime \prime }\right)\right) & =-\sum_{R\in {\overset{⃑}{\tau }}^{\prime }\left(V^{\prime \prime }\right)}\sum_{Q\in {\overset{⃐}{\tau }}^{\prime }\left(V^{\prime }\right)}p\left(Q,R\right)\log p\left(Q|R\right)\\ & =-\sum_{R\in \overset{⃑}{\tau }\left(V^{\prime \prime }\right)}\sum_{Q\in {\overset{⃐}{\tau }}^{\prime }\left(V^{\prime }\right)}p\left(Q,R\right)\log p\left(Q|R\right)-\sum_{Q\in {\overset{⃐}{\tau }}^{\prime }\left(V^{\prime }\right)}p\left(Q,R^{\prime }\right)\log p\left(Q|R^{\prime }\right)\\ & =-\sum_{R\in \overset{⃑}{\tau }\left(V^{\prime \prime }\right)}\left(\sum_{Q\in \overset{⃐}{\tau }\left(V^{\prime }\right)}p\left(Q,R\right)\log p\left(Q|R\right)+p\left(Q^{\prime },R\right)\log p\left(Q^{\prime }|R\right)\right)\\ & \;-\sum_{Q\in \overset{⃐}{\tau }\left(V^{\prime }\right)}p\left(Q,R^{\prime }\right)\log p\left(Q|R^{\prime }\right)-p\left(Q^{\prime },R^{\prime }\right)\log p\left(Q^{\prime }|R^{\prime }\right)\\ & =-\sum_{R\in \overset{⃑}{\tau }\left(V^{\prime \prime }\right)}\sum_{Q\in \overset{⃐}{\tau }\left(V^{\prime }\right)}p\left(Q,R\right)\log p\left(Q|R\right)\\ & \;-\sum_{R\in \overset{⃑}{\tau }\left(V^{\prime \prime }\right)}p\left(Q^{\prime },R\right)\log p\left(Q^{\prime }|R\right)-\sum_{Q\in \overset{⃐}{\tau }\left(V^{\prime }\right)}p\left(Q,R^{\prime }\right)\log p\left(Q|R^{\prime }\right)\\ & \;\;-p\left(Q^{\prime },R^{\prime }\right)\log p\left(Q^{\prime }|R^{\prime }\right). \end{array}$$

In this formula, the first term is equivalent to the conditional entropy of the sets $\overset{⃐}{\tau }\left(V^{\prime }\right)$ and $\overset{⃑}{\tau }\left(V^{\prime \prime }\right)$, the second term represents the influence of the sets $Q^{\prime }$ and $R^{\prime }$ to the elements of the sets $\overset{⃐}{\tau }\left(V^{\prime }\right)$ and $\overset{⃑}{\tau }\left(V^{\prime \prime }\right)$, and the last term defines the conditional entropy of $Q^{\prime }$ with respect to $R^{\prime }$.

This definition is a direct extension of the definition of conditional entropy of the partitions. In fact, if the sets $\overset{⃐}{\tau }\left(V^{\prime }\right)$ and $\overset{⃑}{\tau }\left(V^{\prime \prime }\right)$ are covers of V, then $Q^{\prime }=\emptyset$ and $R^{\prime }=\emptyset$. Then,

$$H\left({\overset{⃐}{\tau }}^{\prime }\left(V^{\prime }\right)|{\overset{⃑}{\tau }}^{\prime }\left(V^{\prime \prime }\right)\right)=H\left(\overset{⃐}{\tau }\left(V^{\prime }\right)|\overset{⃑}{\tau }\left(V^{\prime \prime }\right)\right)=-\sum_{R\in \overset{⃑}{\tau }\left(V^{\prime \prime }\right)}\sum_{Q\in \overset{⃐}{\tau }\left(V^{\prime }\right)}p\left(Q,R\right)\log p\left(Q|R\right),$$

and if $\overset{⃐}{\tau }\left(V^{\prime }\right)$ and $\overset{⃑}{\tau }\left(V^{\prime \prime }\right)$ are partitions of V, then it is equivalent to the definition of the conditional entropy.

Note that since ${\overset{⃐}{\tau }}^{\prime }\left(V^{\prime }\right)$ and ${\overset{⃑}{\tau }}^{\prime }\left(V^{\prime \prime }\right)$ are covers of the set V, the conditional entropy $H\left({\overset{⃐}{\tau }}^{\prime }\left(V^{\prime }\right)|{\overset{⃑}{\tau }}^{\prime }\left(V^{\prime \prime }\right)\right)$ does not necessarily meet all the properties of the conditional entropy defined for the partitions. However, here, we will not consider specific properties of the conditional entropy of the covers but will use it directly to define the distance between the predecessor cover $\overset{⃐}{\tau }\left(V^{\prime }\right)$ and the successor cover $\overset{⃑}{\tau }\left(V^{\prime \prime }\right)$.

The distance $d\left(\overset{⃐}{\tau }\left(V^{\prime }\right),\overset{⃑}{\tau }\left(V^{\prime \prime }\right)\right)$ between the predecessor cover $\overset{⃐}{\tau }\left(V^{\prime }\right)$ and the successor cover $\overset{⃑}{\tau }\left(V^{\prime \prime }\right)$ is defined by the Rokhlin distance between the covers ${\overset{⃐}{\tau }}^{\prime }\left(V^{\prime }\right)$ and ${\overset{⃑}{\tau }}^{\prime }\left(V^{\prime \prime }\right)$ of the set V of vertices as

$$d\left(\overset{⃐}{\tau }\left(V^{\prime }\right),\overset{⃑}{\tau }\left(V^{\prime \prime }\right)\right)=H\left({\overset{⃐}{\tau }}^{\prime }\left(V^{\prime }\right)|{\overset{⃑}{\tau }}^{\prime }\left(V^{\prime \prime }\right)\right)+H\left({\overset{⃐}{\tau }}^{\prime }\left(V^{\prime \prime }\right)|{\overset{⃑}{\tau }}^{\prime }\left(V^{\prime }\right)\right),$$

For example, the distance between the predecessor cover $\overset{⃐}{\tau }\left(V^{c}\right)=\left\{\left\{v_{1},v_{2}\right\},\left\{v_{3},v_{4},v_{8}\right\},\left\{v_{4}\right\},\left\{v_{7},v_{9}\right\}\right\}$ and the successor cover $\overset{⃑}{\tau }\left(V^{c}\right)=\left\{\left\{v_{2},v_{4}\right\},\left\{v_{4}\right\},\left\{v_{5},v_{6}\right\},\left\{v_{5},v_{7}\right\},\left\{v_{8}\right\}\right\}$ is calculated as follows:

The completed sets for the covers $\overset{⃐}{\tau }\left(V^{c}\right)$ and $\overset{⃑}{\tau }\left(V^{c}\right)$ are $Q^{\prime }=\left\{v_{1},v_{2},\ldots ,v_{9}\right\}\backslash \left\{v_{1},v_{2},v_{3},v_{4},v_{7},v_{8},v_{9}\right\}=\left\{v_{5},v_{6}\right\}$ and $R^{\prime }=\left\{v_{1},v_{2},\ldots ,v_{9}\right\}\backslash \left\{v_{2},v_{4},v_{5},v_{6},v_{7},v_{8}\right\}=\left\{v_{1},v_{3},v_{9}\right\}$. Then, the completed covers of the set V of vertices are

$$\overset{⃐}{\tau }\left(V^{c}\right)=\left\{\left\{v_{1},v_{2}\right\},\left\{v_{3},v_{4},v_{8}\right\},\;\left\{v_{4}\right\},\left\{v_{7},v_{9}\right\},\left\{v_{5},v_{6}\right\}\right\}$$

and

$$\overset{⃑}{\tau }\left(V^{c}\right)=\left\{\left\{v_{2},v_{4}\right\},\left\{v_{4}\right\},\left\{v_{5},v_{6}\right\},\left\{v_{5},v_{7}\right\},\;\left\{v_{8}\right\},\left\{v_{1},v_{3},v_{9}\right\}\right\}.$$

The probability of each vertex v∈V is $p\left(v\right)=\frac{1}{9}$. Then, conditional entropies $H\left({\overset{⃐}{\tau }}^{\prime }\left(V^{c}\right)|{\overset{⃑}{\tau }}^{\prime }\left(V^{c}\right)\right)$ and $H\left({\overset{⃑}{\tau }}^{\prime }\left(V^{c}\right)|{\overset{⃐}{\tau }}^{\prime }\left(V^{c}\right)\right)$ are (the zero terms are omitted).

$$\begin{array}{ll} H\left({\overset{⃐}{\tau }}^{\prime }\left(V^{c}\right)|{\overset{⃑}{\tau }}^{\prime }\left(V^{c}\right)\right) & =-p\left(\left\{v_{2}\right\}\right)\log\frac{p\left(\left\{v_{2}\right\}\right)}{p\left(\left\{v_{2},v_{4}\right\}\right)}-p\left(\left\{v_{4}\right\}\right)\log\frac{p\left(\left\{v_{4}\right\}\right)}{p\left(\left\{v_{2},v_{4}\right\}\right)}\\ & \;-p\left(\left\{v_{4}\right\}\right)\log\frac{p\left(\left\{v_{4}\right\}\right)}{p\left(\left\{v_{2},v_{4}\right\}\right)}-p\left(\left\{v_{7}\right\}\right)\log\frac{p\left(\left\{v_{7}\right\}\right)}{p\left(\left\{v_{5},v_{7}\right\}\right)}-p\left(\left\{v_{5}\right\}\right)\log\frac{p\left(\left\{v_{5}\right\}\right)}{p\left(\left\{v_{5},v_{7}\right\}\right)}\\ & \;-p\left(\left\{v_{1}\right\}\right)\log\frac{p\left(\left\{v_{1}\right\}\right)}{p\left(\left\{v_{1},v_{3},v_{9}\right\}\right)}-p\left(\left\{v_{3}\right\}\right)\log\frac{p\left(\left\{v_{3}\right\}\right)}{p\left(\left\{v_{1},v_{3},v_{9}\right\}\right)}-p\left(\left\{v_{9}\right\}\right)\log\frac{p\left(\left\{v_{9}\right\}\right)}{p\left(\left\{v_{1},v_{3},v_{9}\right\}\right)}\\ & =-\frac{1}{9}\log\frac{1}{2}-\frac{1}{9}\log\frac{1}{2}-\frac{1}{9}\log\frac{1}{2}-\frac{1}{9}\log\frac{1}{2}-\frac{1}{9}\log\frac{1}{2}-\frac{1}{9}\log\frac{1}{3}-\frac{1}{9}\log\frac{1}{3}-\frac{1}{9}\log\frac{1}{3}\\ & =1.08, \end{array}$$

$$\begin{array}{ll} H\left({\overset{⃑}{\tau }}^{\prime }\left(V^{c}\right)|{\overset{⃐}{\tau }}^{\prime }\left(V^{c}\right)\right) & =-p\left(\left\{v_{2}\right\}\right)\log\frac{p\left(\left\{v_{2}\right\}\right)}{p\left(\left\{v_{1},v_{2}\right\}\right)}-p\left(\left\{v_{1}\right\}\right)\log\frac{p\left(\left\{v_{1}\right\}\right)}{p\left(\left\{v_{1},v_{2}\right\}\right)}\\ & \;-p\left(\left\{v_{4}\right\}\right)\log\frac{p\left(\left\{v_{4}\right\}\right)}{p\left(\left\{v_{3},v_{4},v_{8}\right\}\right)}-p\left(\left\{v_{4}\right\}\right)\log\frac{p\left(\left\{v_{4}\right\}\right)}{p\left(\left\{v_{3},v_{4},v_{8}\right\}\right)}-p\left(\left\{v_{8}\right\}\right)\log\frac{p\left(\left\{v_{8}\right\}\right)}{p\left(\left\{v_{3},v_{4},v_{8}\right\}\right)}\\ & \;-p\left(\left\{v_{3}\right\}\right)\log\frac{p\left(\left\{v_{3}\right\}\right)}{p\left(\left\{v_{3},v_{4},v_{8}\right\}\right)}-p\left(\left\{v_{7}\right\}\right)\log\frac{p\left(\left\{v_{7}\right\}\right)}{p\left(\left\{v_{7},v_{9}\right\}\right)}-p\left(\left\{v_{9}\right\}\right)\log\frac{p\left(\left\{v_{9}\right\}\right)}{p\left(\left\{v_{7},v_{9}\right\}\right)}\\ & \;-p\left(\left\{v_{5}\right\}\right)\log\frac{p\left(\left\{v_{5}\right\}\right)}{p\left(\left\{v_{5},v_{6}\right\}\right)}\\ & =-\frac{1}{9}\log\frac{1}{2}-\frac{1}{9}\log\frac{1}{2}-\frac{1}{9}\log\frac{1}{3}-\frac{1}{9}\log\frac{1}{3}-\frac{1}{9}\log\frac{1}{3}-\frac{1}{9}\log\frac{1}{3}-\frac{1}{9}\log\frac{1}{2}-\frac{1}{9}\log\frac{1}{2}-\frac{1}{9}\log\frac{1}{2}\\ & =1.26, \end{array}$$

The distance between the predecessor and successor covers of the set $V^{c}=\left\{v_{1},v_{4},v_{5},v_{8}\right\}$ of vertices is

$$d\left(\overset{⃐}{\tau }\left(V^{c}\right),\overset{⃑}{\tau }\left(V^{c}\right)\right)=1.08+1.26=2.34.$$

For comparison, the distance between the indicated above predecessor cover $\overset{⃐}{\tau }\left(v_{8}\right)=\left\{\left\{v_{1},v_{2}\right\},\left\{v_{4}\right\},\left\{v_{7},v_{9}\right\}\right\}$ and the successor cover $\overset{⃑}{\tau }\left(v_{8}\right)=\left\{\left\{v_{5},v_{6}\right\},\left\{v_{6}\right\}\right\}$ of the vertex $v_{8}$ is

$$d\left(\overset{⃐}{\tau }\left(v_{8}\right),\overset{⃑}{\tau }\left(v_{8}\right)\right)=1.96.$$

Then, the group $A^{c}=\left\{a_{1},a_{4},a_{5},a_{8}\right\}$ of the agents associated with the vertices of the group $V^{c}=\left\{v_{1},v_{4},v_{5},v_{8}\right\}$ is preferable as a group of leaders than a group $A^{c}=\left\{a_{8}\right\}$, which includes only one agent $a_{8}$ associated with the vertex $v_{8}$.

Hereby, we defined the group $A^{*}\subset A$ of leading agents and suggested the criterion for its recognition among the other agents in the group A. The same procedure can be continued over the group $A^{*}$ and then recurrently over the obtained groups up to distinguishing a unique leading agent.

The algorithmic solution to the problem of distinguishing the leading agents is a complex task which requires an exhaustive search among all possible subsets of the agents from the group A or, that is, the same, among all possible subsets of vertices from the set V. Together with that, certain heuristics omitting the vertices with a relatively small number of predecessors and successors can strongly decrease the number of candidate solutions.

## 5. Distinguishing the Group of Experts

Assume that the group of agents $A=\left\{a_{1},a_{2},\ldots ,a_{l}\right\}$ considers the set $X=\left\{x_{1},\ldots ,x_{n}\right\}$ of n items and each agent $a_{k}\in A$, k=1,2,…,l, provides partition $\gamma_{k}=\left\{C_{k,1},C_{k,2},\ldots ,C_{k,m}\right\}$ of the set X to m classes. The resulting classification is an aggregated partition $\gamma =\left\{C_{1},C_{2},\ldots ,C_{m}\right\}$ created from the agents’ partitions $\gamma_{k}$, k=1,2,…,l, and to obtain the correct partition γ, it is required to recognize partitions provided by the competent agents and avoid partitions provided by non-competent agents.

Distinguishing the experts is based on the assumption that the agents with the same competence in the same fields provide similar classifications of the items related to their field of expertise and can provide different classifications of the items that are outside of the scope of their competence [12]. In other words, we follow the well-known phrase by Father Dominique Bouhours ([28] (p. 125), punctuation and grammar preserved):

*“Great Minds often think alike on the same Occasions, and we are not always to suppose, that such Thoughts are borrow’d from one another when exprest by Persons of the same heroick Sentiments.”*

Following this assumption, agent $a_{k}\in A$ is considered a weak expert in a certain class $C_{j}$ if the agent’s partition $\gamma_{k}$ includes $C_{j}$ and there exist the other agents $a_{k^{\prime }},a_{k^{\prime \prime }},\ldots \in A$, such that their partitions $\gamma_{k^{\prime }}$, $\gamma_{k^{\prime \prime }}$,… include $C_{j}$. If the partition $\gamma_{k}$ class $C_{j}$ is at the same position as in the partitions $\gamma_{k^{\prime }}$, $\gamma_{k^{\prime \prime }}$,…, then the agent $a_{k}$ is called strong expert or expert, for briefness.

The number of agents with equivalent classes $C_{j}$ required for specifying the agent as an expert varies and depends on the number l of agents in the group. Following general statistical assumptions, we say that the number of such agents is at least 10% of l and, for small groups, is not less than 2.

As indicated above, there exist several algorithms of classification that implement the difference between the opinions of competent and non-competent agents [10,11,12,13]. In particular, the Distance-Based Collaborative Classification (DBCC) algorithm [12] directly considers the normalized Hamming distance $d_{H}\left(\gamma_{k},\gamma_{k^{\prime }}|j\right)$ between the partitions $\gamma_{k}=\left\{C_{k,1},C_{k,2},\ldots ,C_{k,m}\right\}$ and $\gamma_{k^{\prime }}=\left\{C_{k^{\prime },1},C_{k^{\prime },2},\ldots ,C_{k^{\prime },m}\right\}$ with respect to each class $C_{j}$

$$d_{nH}\left(\gamma_{k},\gamma_{k^{\prime }}|j\right)=\#\left(C_{k,j}∆C_{k^{\prime },j}\right)/\left(\#C_{k,j}+\#C_{k^{\prime },j}\right),$$

where $C_{k,j}∆C_{k^{\prime },j}=\left(C_{k,j}\cup C_{k^{\prime },j}\right)\backslash \left(C_{k,j}\cap C_{k^{\prime },j}\right)$ is a symmetric difference between the classes $C_{k,j}$ and $C_{k^{\prime },j}$.

If on the set X of items, a probability measure $p:X\to \left[0,1\right]$ is defined, then using this measure, the distance between the partitions $\gamma_{k}$ and $\gamma_{k^{\prime }}$ of X with respect to the class $C_{j}$ can be defined as

$$d_{pH}\left(\gamma_{k},\gamma_{k^{\prime }}|j\right)=-p\left(C_{k,j}∆C_{k^{\prime },j}\right),$$

or in the form of the Rokhlin metric as

$$d_{pR}\left(\gamma_{k},\gamma_{k^{\prime }}|j\right)=-p\left(C_{k,j}\backslash C_{k^{\prime },j}\right)\log p\left(C_{k,j}\backslash C_{k^{\prime },j}\right)-p\left(C_{k^{\prime },j}\backslash C_{k,j}\right)\log p\left(C_{k^{\prime },j}\backslash C_{k,j}\right).$$

Below, we assume that the experts are already distinguished by these or other methods and consider the relationship between the group of leaders and the group of experts.

## 6. Relationship between the Group $A^{*}$ of Leaders and the Group A′ of Experts

Let us return to the above example and assume that the distinguished group of experts is $A^{\prime }=\left\{a_{1},a_{2},a_{3},a_{4},a_{5},a_{6}\right\}$. Our aim is to check whether this group of experts is also a group of leaders.

Denote by $V^{\prime }\subset V$, the set of vertices in the graph G associated with the agents from the group $A^{\prime }$ of experts. Then, following the presented above procedure of distinguishing the group of leading agents, the distance between the predecessor partition $\overset{⃐}{\tau }\left(V^{\prime }\right)$ and the successor partition $\overset{⃑}{\tau }\left(V^{\prime }\right)$ is

$$d\left(\overset{⃐}{\tau }\left(V^{\prime }\right),\overset{⃑}{\tau }\left(V^{\prime }\right)\right)=H\left({\overset{⃐}{\tau }}^{\prime }\left(V^{\prime }\right)|{\overset{⃑}{\tau }}^{\prime }\left(V^{\prime }\right)\right)+H\left({\overset{⃑}{\tau }}^{\prime }\left(V^{\prime }\right)|{\overset{⃐}{\tau }}^{\prime }\left(V^{\prime }\right)\right)=0.89+0.70=1.59.$$

It is seen that the predecessor partition $\overset{⃐}{\tau }\left(V^{\prime }\right)$ and successor partition $\overset{⃑}{\tau }\left(V^{\prime }\right)$ of the set $V^{\prime }$ are closer than the predecessor partition $\overset{⃐}{\tau }\left(V^{c}\right)$ and successor partition $\overset{⃑}{\tau }\left(V^{c}\right)$ of the previously distinguished set $V^{c}=\left\{v_{1},v_{4},v_{5},v_{8}\right\}$ of vertices associated with the agents from the set $A^{c}$ of candidate leaders (distance $d\left(\overset{⃐}{\tau }\left(V^{c}\right),\overset{⃑}{\tau }\left(V^{c}\right)\right)=2.34$). Moreover, partitions $\overset{⃐}{\tau }\left(V^{\prime }\right)$ and $\overset{⃑}{\tau }\left(V^{\prime }\right)$ are closer than the partitions $\overset{⃐}{\tau }\left(v_{8}\right)$ and $\overset{⃑}{\tau }\left(v_{8}\right)$ of the vertex $v_{8}$ (distance $d\left(\overset{⃐}{\tau }\left(v_{8}\right),\overset{⃑}{\tau }\left(v_{8}\right)\right)=1.96$).

Now, let us consider the classifications provided by the candidate group $A^{c}=\left\{a_{1},a_{4},a_{5},a_{8}\right\}$ of leaders. We have

$$\gamma_{1}=\left\{\left\{x_{2},x_{6},x_{8}\right\},\left\{x_{1}\right\},\left\{x_{3},x_{9},x_{10},x_{12}\right\},\left\{x_{4},x_{5},x_{7},x_{11}\right\}\right\},\;\gamma_{4}=\left\{\left\{x_{3},x_{6},x_{7},x_{10},x_{12}\right\},\left\{x_{8},x_{9}\right\},\left\{x_{4},x_{5},x_{11}\right\},\left\{x_{1},x_{2}\right\}\right\},\;\gamma_{5}=\left\{\left\{x_{3},x_{4},x_{10}\right\},\left\{x_{5},x_{6},x_{8},x_{11}\right\},\left\{x_{1},x_{2}\right\},\left\{x_{7},x_{9},x_{12}\right\}\right\},\;\gamma_{8}=\left\{\left\{x_{3},x_{10}\right\},\left\{x_{2},x_{8}\right\},\left\{x_{1},x_{4},x_{5},x_{6},,x_{11},x_{12}\right\},\left\{x_{7},x_{9}\right\}\right\}.$$

Despite the competence of agent $a_{1}$ in class $C_{1}$ of agent $a_{4}$ in class $C_{3}$ and of agent $a_{5}$ in class $C_{5}$, the resulting plurality voting partition is (item $x_{2}$ is labeled randomly)

$$\gamma_{Pl}=\left\{\left\{x_{3},x_{6},x_{10}\right\},\left\{x_{2},x_{8}\right\},\left\{x_{1},x_{5},x_{11},x_{12}\right\},\left\{x_{4},x_{7},x_{9}\right\}\right\},$$

which is far from the correct partition $\overset{ˇ}{\gamma }$.

Thus, in the considered example with random relations between the agents $a_{k}$ and $a_{k^{\prime }}$ and randomly chosen classifications $\gamma_{k}$, $k,k^{\prime }=1,2,\ldots ,l$, the group of experts strongly differs from the group of leading agents.

However, as indicated above, it is reasonable to assume that the agent elected to be a leader or a member of the group of leaders is competent in certain fields [19].

Following this assumption, let us consider the other example and form a group of leaders starting with the agents’ expertise [13]. Assume that the group A of l=8 agents classifies n=9 items x from the set X over m=5 classes C. The agents’ classifications are shown in Table 3.

By the Wisdom in the Crowd (WICRO) algorithm [13], the agents are divided into clusters based on the number of agreements about the classes for each item x. The clusters obtained by the agents are summarized in Table 4.

Following the table, $a_{1}$ and $a_{2}$ are the agents that agree that the item $x_{1}$ should be in the class $C_{1}$ and the item $x_{3}$ should be in the class $C_{3}$; $a_{1}$, $a_{2}$, and $a_{7}$ are the agents that agree that the item $x_{2}$ should be in the class $C_{2}$ and so on.

In addition, it is seen that the agents $a_{1}$ and $a_{2}$ appear both in the cluster $\left\{a_{1},a_{2}\right\}$ and in the cluster $\left\{a_{1},a_{2},a_{7}\right\}$ together with the agent $a_{7}$. Thus, we assume that the agents $a_{1}$ and $a_{2}$ are predecessors and successors of each other and both are predecessors of the agent $a_{7}$. The same holds for the agents $a_{5}$ and $a_{6}$ and the agent $a_{4}$.

Also, the agents $a_{3}$ and $a_{4}$ appear in two clusters $\left\{a_{1},a_{3},a_{4}\right\}$ and $\left\{a_{2},a_{3},a_{4}\right\}$, while each of the agents $a_{1}$ and $a_{2}$ appear in only one of these two clusters. So, we assume that the agents $a_{3}$ and $a_{4}$ are predecessors and successors of each other and both are predecessors of the agents $a_{1}$ and $a_{2}$.

Finally, the agent $a_{8}$ does not appear in any cluster, so we assume that this agent is a successor of all other agents.

**Figure 3:**
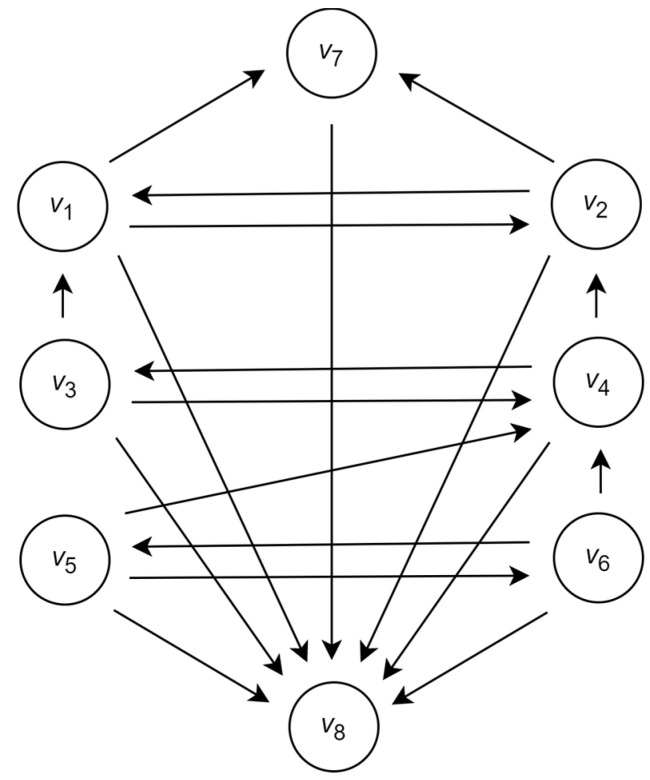
Graph of communication between the agents with respect to the agents’ clustering.

Associating the agents with the vertices and the relations between the agents with the edges, one obtains the directed graph $G=\left(V,E\right)$ shown in Figure 3.

Similarly to Table 1, the sets of input and output vertices in this graph are presented in Table 5.

Following this graph, the clear leader is the agent $a_{4}$ associated with the vertex $v_{4}$ and additional leaders are the agents $a_{1}$ and $a_{2}$ associated with the vertices $v_{1}$ and $v_{2}$. Together with that, according to the structure of the graph, none of the agents is a dictator or monarch.

The further application of the majority voting in the group $A^{*}=\left\{a_{1},a_{2},a_{4}\right\}$ of leading agents results in the classification that is correct for items $x_{1}$, $x_{2}$, $x_{3}$ and $x_{5}$, is incorrect for items $x_{6}$, $x_{7}$ and $x_{8}$, and with probability $\frac{1}{3}$ can be correct for each of the items $x_{4}$ and $x_{9}$.

## 7. Conclusions

The paper considered the problem of distinguishing the leaders in the group of autonomous agents.

In the paper, we suggested a definition of the leading agents, which are the agents that maximally divide the group. For calculating the distances between the subgroups of the agents, we use the entropy-based Rokhlin metric, which was extended for measuring the distances between the covers of the sets.

In the framework of classification problems, the paper considers the relationship between the competent agents and the leading agents and presents an example of distinguishing the leaders based on their expertise in certain fields of knowledge.

The suggested method can be used in programming the division of labor in the swarm activity dynamics and in the analysis of the data fusion in the records obtained by the wisdom of the crowd techniques.

Further research will include verification of the method on a wider range of data and consideration of the relations between the properties of the graphs, the groups of the distinguished leading agents, and the levels of their expertise.

## Figures and Tables

**Table 1:** The sets of input and output vertices in the graph G.

Vertex	$v_{1}$	$v_{2}$	$v_{3}$	$v_{4}$	$v_{5}$	$v_{6}$	$v_{7}$	$v_{8}$	$v_{9}$
Input set	∅	$\left\{v_{1}\right\}$	∅	$\left\{v_{1},v_{2}\right\}$	$\left\{v_{4},v_{4},v_{8}\right\}$	$\left\{v_{5},v_{8}\right\}$	$\left\{v_{4}\right\}$	$\left\{v_{7},v_{9}\right\}$	∅
Output set	$\left\{v_{1},v_{4}\right\}$	$\left\{v_{4}\right\}$	$\left\{v_{5}\right\}$	$\left\{v_{5},v_{7}\right\}$	$\left\{v_{6}\right\}$	∅	$\left\{v_{8}\right\}$	$\left\{v_{5},v_{6}\right\}$	$\left\{v_{8}\right\}$

**Table 2:** Example of n=12 items distributed by l=9 agents over m=4 classes. Partitions $\gamma_{k}$, k=1,2,…,9 represent the agents’ classifications; partition $\overset{ˇ}{\gamma }$ represents correct classification; and partition $\gamma_{Pl}$ represents the result of plurality voting.

	$\gamma_{1}$	$\gamma_{2}$	$\gamma_{3}$	$\gamma_{4}$	$\gamma_{5}$	$\gamma_{6}$	$\gamma_{7}$	$\gamma_{8}$	$\gamma_{9}$	$\overset{ˇ}{\gamma }$	$\gamma_{Pl}$
$x_{1}$	2	2	2	4	3	1	1	3	4	2	2
$x_{2}$	1	3	1	4	3	1	1	2	3	1	1
$x_{3}$	3	2	2	1	1	1	2	1	3	2	1
$x_{4}$	4	4	4	3	1	3	4	3	1	3	4
$x_{5}$	4	4	4	3	2	3	4	3	2	3	4
$x_{6}$	1	3	1	1	2	2	2	3	1	1	1
$x_{7}$	4	3	3	1	4	4	4	4	3	4	4
$x_{8}$	1	3	1	2	2	2	1	2	3	1	2
$x_{9}$	3	3	4	2	4	4	2	4	4	4	4
$x_{10}$	3	2	2	1	1	1	2	1	1	2	1
$x_{11}$	4	3	4	3	2	3	4	3	3	3	3
$x_{12}$	3	3	3	1	4	4	1	3	4	4	3

**Table 3:** Example of n=9 items distributed by l=8 agents over m=5 classes. Partitions $\gamma_{k}$, k=1,2,…,8 represent the agents’ classifications and partition $\overset{ˇ}{\gamma }$ represents correct classification.

	$\gamma_{1}$	$\gamma_{2}$	$\gamma_{3}$	$\gamma_{4}$	$\gamma_{5}$	$\gamma_{6}$	$\gamma_{7}$	$\gamma_{8}$	$\overset{ˇ}{\gamma }$
$x_{1}$	1	1	2	4	3	5	4	3	1
$x_{2}$	2	2	3	4	3	4	2	5	2
$x_{3}$	3	3	2	1	4	2	5	2	3
$x_{4}$	5	1	4	4	3	3	2	1	4
$x_{5}$	5	2	5	5	1	4	4	3	5
$x_{6}$	5	2	2	2	3	1	1	4	1
$x_{7}$	4	3	1	5	2	2	4	4	2
$x_{8}$	2	1	5	2	3	2	1	2	3
$x_{9}$	5	1	2	4	4	4	3	2	4

**Table 4:** Clusters of the agents with respect to the number of agreements about the classes of the items.

	Agent’s Cluster	Chosen Class Number
$x_{1}$	$\left\{a_{1},a_{2}\right\}$	1
$x_{2}$	$\left\{a_{1},a_{2},a_{7}\right\}$	2
$x_{3}$	$\left\{a_{1},a_{2}\right\}$	3
$x_{4}$	$\left\{a_{5},a_{6}\right\}$	3
$x_{5}$	$\left\{a_{1},a_{3},a_{4}\right\}$	5
$x_{6}$	$\left\{a_{2},a_{3},a_{4}\right\}$	2
$x_{7}$	$\left\{a_{5},a_{6}\right\}$	2
$x_{8}$	$\left\{a_{5},a_{6}\right\}$	3
$x_{9}$	$\left\{a_{4},a_{5},a_{6}\right\}$	4

**Table 5:** The sets of input and output vertices in the graph G shown in Figure 3.

Vertex	$v_{1}$	$v_{2}$	$v_{3}$	$v_{4}$	$v_{5}$	$v_{6}$	$v_{7}$	$v_{8}$
Input set	$\left\{v_{2},v_{3}\right\}$	$\left\{v_{1},v_{4}\right\}$	$\left\{v_{4}\right\}$	$\left\{v_{3},v_{5},v_{6}\right\}$	$\left\{v_{6}\right\}$	$\left\{v_{5}\right\}$	$\left\{v_{1},v_{2}\right\}$	∅
Output set	$\left\{v_{2},v_{7},v_{8}\right\}$	$\left\{v_{1},v_{7},v_{8}\right\}$	$\left\{v_{1},v_{4},v_{8}\right\}$	$\left\{v_{2},v_{3},v_{8}\right\}$	$\left\{v_{4},v_{6},v_{8}\right\}$	$\left\{v_{4},v_{5},v_{8}\right\}$	$\left\{v_{8}\right\}$	$V\backslash \left\{v_{8}\right\}$

## Data Availability

No new data were created or analyzed in this study. Data sharing is not applicable to this article.
